# Butyric, lactic, and propionic acids with their salts as natural growth promoters in broilers

**DOI:** 10.1038/s41598-025-26549-1

**Published:** 2025-11-20

**Authors:** Ahmed Samy, Hany M. R. Elsherif

**Affiliations:** 1 Department of Animal Production, National Research Centre, Cairo, 12622 Egypt; 2 Animal Production Department, Faculty of Agriculture, Cairo University, Giza, Egypt

**Keywords:** Organic acids, Salts, Broilers, Productive performance, Humoral immunity, Intestinal microbiota, Biochemistry, Biotechnology, Immunology, Microbiology

## Abstract

This study evaluates the effects of lactic, propionic and butyric acids, along with their salts, as natural antibiotic alternatives and growth promoters in broiler diets. Three hundred one-day-old Cobb broiler chicks (*n* = 60 per group), with six replicates of 10 birds each, were randomly allocated to five dietary treatments for 35 days: T1 (control, basal diet); T2 (basal diet + lactic acid and lactate, 0.5 g/kg each); T3 (basal diet + propionic acid and propionate, 0.5 g/kg each); T4 (basal diet + butyric acid and butyrate, 0.5 g/kg each); and T5 (basal diet + a mixture of lactic, propionic, and butyric acids and their salts, 0.17 g/kg each; total ≈ 1 g/kg). Growth performance, blood biochemistry, antioxidant indicators, thyroid hormones, carcass characteristics, intestinal microbiota, and humoral immune responses were assessed. The findings indicate that the blend of organic acids and their salts significantly improved (*p* ≤ 0.05) productive performance during the grower, finisher, and overall periods. Serum antioxidant indices (TAC, CAT) and RBC enzymatic activities (SOD, CAT) exhibited significant increases (*p* ≤ 0.05), whereas MDA levels were significantly decreased (*p* ≤ 0.05). T4 hormone levels increased (*p* ≤ 0.05) in the treated groups without affecting liver and kidney functions. The intestinal microbiota exhibited elevated (*p* ≤ 0.05) *Lactobacillus* levels and inhibited *E. coli* proliferation. Humoral immunity exhibited significantly higher (*p* ≤ 0.05) HI titers against avian influenza viruses (H9 and H5) than the control group. Overall, supplementation with organic acids and their salts offers a suitable natural growth promoter that enhances broiler productive performance, immunity, and antioxidant status without adversely affecting physiological functions.

## Introduction

The growing demand for an effective source of animal protein worldwide has led to a major increase of the poultry sector in recent decades^1^. So, growth promoters are used to improve poultry productive performance and feed efficiency^2–5^. Historically, broiler diets have frequently included antibiotics as growth promoters^6^. The misuse of antibiotics, it led to antimicrobial resistance, which poses considerable risks to both animals and their consumers^7,8^.

Consequently, the European Union outlawed antibiotics as growth enhancers in broiler diets, and many nations severely limited their usage. Therefore, the scientists looked for a natural and efficient substitute for antibiotics as growth enhancers to improve broiler performance without any adverse effects on food safety or animal health^9–11^.

Among the most promising possibilities are organic acids (OA) and their derivatives. These have an antibacterial effect, enhancing the helpful bacteria, and inhibiting the harmful bacteria. In addition, it is acidifying and important for gut integrity^12,13^. The gastrointestinal system’s pH can be lowered by organic acids like lactic, propionic, and butyric acids, which helps to increase beneficial bacteria like *Lactobacillus* species and decrease pathogenic bacteria^14^. Furthermore, their salts improve palatability, stability, and solubility, rendering them more appropriate for incorporation into poultry feeds^13^.

Lactic acid and its salts inhibit bacterial colonization and support epithelial integrity, leading to enhanced gut health. Butyric acid provides energy for gut cells and helps improve the intestinal morphology, while propionic acid effectively slows down the proliferation of *Salmonella* and *E. coli* bacteria^15^. These acids could have synergistic effects, thereby enhancing their respective advantages^16^.

Previous studies of dietary supplements, including OA, have demonstrated an enhancement in broiler productivity, a boost in nutrient digestibility, alteration of immunological responses, and lower oxidative state^16,17^. However, comparative studies comparing single against combination organic acid-salt supplementation across a wide range of physiological and immunological criteria, remain few^18^.

The present study therefore sought to assess, individually and in combination, lactic, propionic, and butyric acids and their salts in improving growth performance, thyroid function, blood biochemistry, antioxidant status, carcass traits, intestinal microbial balance, and humoral immune responses in broiler chickens. The findings aim to contribute to the growing evidence supporting the safe and sustainable replacement of antibiotics in broiler diets with organic acids.

## Materials and methods

### Animal welfare and ethical approval

The National Research Centre Medical Research Ethics Committee approved the animal experiment (Approval Number: 13050405). The research adhered to the International Health Organization’s rules, Institutional Animal Care and Use Committee (IACUC), Good Medical and Laboratory Practice, recommendations, and relevant Egyptian legislation. European Union (EU) rules were observed in the care and use of the animals, including the ARRIVE guidelines (https://arriveguidelines.org/).

### Anesthesia and euthanasia statement

All animal treatments were performed in compliance with the veterinarian’s best practices. Anesthesia was initiated using a mixture of ketamine (20–40 mg/kg) and xylazine (2–4 mg/kg) to guarantee sufficient drowsiness and analgesia. Euthanasia was conducted via CO₂ inhalation, in accordance with the American Veterinary Medical Association (AVMA) Guidelines for the Euthanasia of Animals (2020). This technique guarantees a humane and painless demise by producing hypoxia, resulting in unconsciousness followed by euthanasia.

### Materials

The organic acids (Lactic, propionic or butyric acids) used were food-grade, commercially available products sourced from certified manufacturers, while their salts were prepared.

### Salt of organic acids preparation

A Solution of 0.1 N of Ca(OH)₂ was gradually added drop by drop to the organic acid (lactic, propionic, or butyric acid) while mixing until the pH was neutral (around 7). Continue stirring for 10–15 min to ensure the reaction reaches its full completion. Obtain the calcium compound (lactate, propionate, or butyrate) as a white crystalline or powdery solid by gentle heating under reduced pressure. Dry the final product in the oven^2^.

$$2{\text{R-COOH}}\,+\,{\text{Ca}}{\left( {{\text{OH}}} \right)_2} \to {\left( {{\text{R-COO}}} \right)_2}{\text{Ca }}+{\text{ }}2{{\text{H}}_2}{\text{O}}$$

### The experiment site

In Giza, Egypt, inside Cairo University’s Agriculture College, is the Poultry Nutrition Research Unit (PNRU), which served as the site for the field experiment. All laboratory analyses were conducted at Egypt’s National Research Centre.

### Experimental design and bird management

At one day of age, 300 male Cobb-500 broiler chicks were purchased from a commercial hatchery. When they arrived, chicks were individually weighed and then assigned at random to five experimental feeding groups (*n* = 60 per group), each with six replicates (10 birds per replication). Under normal hygienic standards and ventilation systems, fresh wood shavings were used in the floor pens where the birds were housed. All pens were part of the same environmentally controlled facility.

Temperature, and humidity were kept per Cobb-500 management recommendations throughout the 35-day study period. Birds have had unrestricted access to feed and fresh water. Approved by the local ethics committee, all animal-handling techniques followed institutional and national recommendations for the ethical treatment of animals in research.

Following established protocols for poultry management and the guidelines laid out by the European Union Directive 2010/63/EU regarding the protection of animals utilized in research, the birds in the experiment were kept under a lighting schedule consisting of 16 h of light and 8 h of darkness each day.

### Dietary treatments

For each growth phase; starter (1–12 d), grower (13–26 d), and finisher (27–35 d); a basal diet consisting of corn and soybean meal was created to meet the Cobb-500 guideline’s recommended nutritional requirements, as illustrated in Table 1.

**Table 1:** Formulation and nutrients composition of the experimental diets.

Ingredients %	Period		
	Starter (1–12 days)	Grower (13–26 days)	Finisher (27–35 days)
Yellow maize	55.55	59.13	64.05
46% soybean meal	39.50	36.40	30.90
Soybean oil	0.60	1.00	1.00
Wheat bran	0.00	0.00	0.90
Limestone	1.60	1.20	1.10
Mono calcium phosphate	1.12	0.80	0.55
Salt (NaCl)	0.24	0.26	0.26
NaHCO_3_	0.24	0.22	0.22
Vitamin and mineral mix^(1)^	0.30	0.30	0.30
DL-methionine	0.24	0.22	0.21
L-lysine HCl	0.24	0.15	0.19
Threonine	0.10	0.05	0.05
Toxin binder	0.10	0.10	0.10
Coccidiostat	0.05	0.05	0.05
Phytase	0.01	0.01	0.01
Energy enzymes	0.01	0.01	0.01
Choline chloride	0.10	0.10	0.10
Total	100	100	100
Calculated composition^(2)^			
Crude protein %	23	21.50	19.5
ME (kcal/kg)	2900	2975	3025
Ether extract%	3.01	3.61	3.77
Crude fiber%	2.77	2.72	2.70
Methionine %	0.55	0.51	0.48
Lysine %	1.23	1.18	1.08
Methionine + cystine %	0.86	0.81	0.76
Threonine %	0.88	0.79	0.72
Calcium %	0.93	0.72	0.65
Nonphytate P %	0.40	0.32	0.26
Sodium%	0.18	0.18	0.18
Chlorine%	0.23	0.23	0.23

^(1)^Per kg of diet, the following vitamin-mineral mixture is provided: Vitamin A, 12,000 IU; Vitamin E, 10 mg; Vitamin D3, 2200 IU; Vitamin K3, 2 mg; Vitamin B1, 1 mg; Vitamin B6, Vit B2, 4 mg; 1.5 mg; Vitamin B12, 10 mg; Pantothenic acid, 10 mg; Niacin, 20 mg; Folic acid, 1 mg; Biotin, 50 mg; Choline chloride, 500 mg; Iodine, 1 mg; Copper, 10 mg; Iron, 30 mg; Selenium, 0.1 mg; Zinc, 50 mg and Manganese, 55 mg.

^(2)^According to NRC 1994.

The experimental design included five dietary treatments: T1 (control, basal diet without additives); T2 (control diet supplemented with 0.5 g/kg lactic acid and 0.5 g/kg lactate); T3 (control diet supplemented with 0.5 g/kg propionic acid and 0.5 g/kg propionate); T4 (control diet supplemented with 0.5 g/kg butyric acid and 0.5 g/kg butyrate); and T5 (control diet supplemented with a mixture of organic acids and their salts, each at 0.17 g/kg, providing lactic acid + lactate, propionic acid + propionate, and butyric acid + butyrate, with a total addition of approximately 1 g/kg diet). The dietary dose of 1 g/kg feed was selected based on previous reports indicating effective ranges of 0.5–2 g/kg for organic acids and their salts in broiler diets^19–21^. This level was considered optimal to balance efficacy and safety and is consistent with commercial recommendations for practical poultry nutrition^22^.

### Growth performance

On days 1, 12, 26, and 35 each individual body weight was noted. Feed refusal was subtracted from the total supply to determine the feed intake for each replicate. The following calculations were done: increase in weight (g) and feed conversion ratio (FCR = g feed / g weight gain).

### Blood biochemical parameters, oxidative Indicators, and immune responses

Thirty blood samples, six birds per treatment, one per replicate, were randomly chosen at day 35 and blood samples were obtained using sterile syringes from jugular venipuncture. Two aliquots from blood were separated on plain tubes: One for serum separation, ten minutes at 3000 rpm in a centrifuge and another for fraction analysis of red blood cells (RBC).

The following parameters were examined:

Using commercial kits (BioMed Diagnostics, Germany), hepatic and renal functions: ALT, AST, and creatinine; Total protein: used biuret technique^23,24^.

Thyroid hormones: T3 and T4 levels obtained with ELISA kits (MyBioSource Inc., USA).

### Signs of oxidative stress

Malondialdehyde (MDA), catalase (CAT) and Total antioxidant capacity (TAC) serum equivalents^25,26^.

In RBCs: CAT and superoxide dismutase (SOD).

A total of 6 samples per treatment were analyzed, and each sample was measured in duplicate using a UV-Vis spectrophotometer (Shimadzu, Japan).

Insulin like growth factor: IGF1 concentration was determined in serum using ELISA Kits (Mybiosource).

### Evaluating immune response

Immunoglobulins (IgM and IgG) levels were measured in serum using ELISA kits (Mybiosource) under normal conditions without any stress^27^.

Hemagglutination inhibition (HI) tests against Newcastle Disease Virus (NDV) and Avian Influenza H9 and H5 strains detected serum antibody titers^28^.

### Microbiota of intestines

At day 35, cecal contents were aseptically collected from six birds per treatment. Serial dilutions were prepared and plated on selective agar media: de Man, Rogosa and Sharpe (MRS) agar for *Lactobacillus* spp. under anaerobic conditions, Eosin Methylene Blue (EMB) agar for *Escherichia coli*, and Plate Count Agar (PCA) for total viable counts. Plates were incubated at 37 °C for 48 h, and bacterial counts were expressed as log10 colony-forming units (CFU)/g of cecal content^29^.

### Carcass traits

At the end of the experiment, two birds per replicate (12 birds per treatment), which were the same birds used for blood sampling, were slaughtered under humane slaughter techniques. Digital balance with ± 0.01 g precision recorded weights. Dressing percentage was calculated as (eviscerated carcass/live body weight × 100) and the relative weights of the bursa of Fabricius, gizzard, liver, spleen, and heart were expressed as percentage of live body weight. Intestinal length measured from duodenum to cloaca.

### Statistical analysis

One-way ANOVA in SAS program (version 9.4)^30^ was employed to analyze all of the data. Duncan multiple range test^31^ helped to investigate variations in treatment means. A p-value of less than 0.05 indicates statistical significance. Means ± standard error was used to report the results.

Statistical model: Y_ij_ = µ + T_i_ + E_ij_, Where Y_ij_ is the observation of the parameter measured, µ is the overall mean; T_i_; is the fixed effect of the treatment and E_ij_ is random error.

## Results

### Growth performance

Table 2 demonstrates that incorporating of organic acids (lactic, propionic, and butyric acids) and their salts substantially influenced broiler growth performance at various stages. In the starter phase (1–12 days), no statistically significant difference (*p* > 0.05) was seen in BWG or FCR among the groups. In the grower phase (12–26 days), all groups that had birds fed diets containing organic acids and their salts had a superior improvement (*p* ≤ 0.05) in FCR and BWG than the control. The highest WG results were seen in birds that ate diets with lactic acid or butyric acid and their salts or the combination of all three acid-salt mixtures, showing weights of 943, 1000, or 993 g, respectively. Birds that ate propionic acid and its salt gained 894 g, while the control group only gained 815 g, showing that these diets helped the birds use their feed more efficiently.

**Table 2:** The impact of dietary interventions on productive performance.

	Item	Control	Lactic+lactate	Propionic+propionate	Butyric+butyrate	Combination of them	SE	Significances
Starter (1–12 day)	BWG (g)	325	364	363	333	344	± 12.21	NS
	FI (g)	425	455	454	417	431	± 16.39	NS
	FCR g feed/ g gain	1.31	1.25	1.25	1.25	1.25	± 0.01	NS
Grower (12–26 day)	BWG (g)	815^c^	943^a^	894^b^	1000^a^	993^a^	± 24.29	***
	FI (g)	1517	1485	1400	1586	1570	± 45.44	NS
	FCR g feed/ g gain	1.86^a^	1.57^b^	1.56 ^b^	1.59 ^b^	1.58 ^b^	± 0.01	***
Finisher (27–35 day)	BWG (g)	674^b^	817^a^	759^a^	742^a^	756^=^	± 28.57	**
	FI (g)	1233	1336	1270	1255	1243	± 32.66	NS
	FCR g feed/ g gain	1.83^a^	1.64 ^b^	1.67 ^b^	1.69 ^b^	1.65 ^b^	± 0.03	**
Overall (1–35 day)	BWG (g)	1814^b^	2101^a^	1975 ^a^	2076 ^a^	2092 ^a^	± 63.12	**
	FI (g)	3175	3276	3125	3257	3245	± 80.81	NS
	FCR g feed/ g gain	1.69^a^	1.56 ^b^	1.59 ^b^	1.57 ^b^	1.55 ^b^	± 0.01	***

At the 0.05 level of probability, means that have the same letter inside the same column are not statistically different; **P < 0.01,***P < 0.001, NS: Not significant (*P* > 0.05).

In the finisher phase (26–35 days), supplementation of OA + S, or the combination of all three acid-salt mixtures, in the broiler diets led to significantly enhanced (*p* ≤ 0.05) productive performance (FCR and BWG) than the control.

Throughout all development periods, no significant change (*p* > 0.05) in feed intake was noted between the control and treatment groups.

During the 35 days, birds that ate diets with added lactic, propionic, or butyric acid and their salts, or the combination of all three acid-salt mixtures, significantly (*p* ≤ 0.05) gained more weight (2101, 1975, 2076, and 2092 g, respectively) than the control group, which gained 1814 g. Furthermore, their FCR was enhanced to 1.56, 1.59, 1.57, and 1.55, in contrast to the control value of 1.69.

### Blood parameters

The impact of OA + S on blood biochemical parameters is presented in Table 3.

**Table 3:** Dietary interventions’ impact on blood markers at 35 days of age.

Item	Total protein (g/dl)	Creatinine (mg/dl)	ALT (U/L)	AST (U/L)
Control	3.31	0.58	15.43	183
Lactic + lactate	3.53	0.53	14.53	176
Propionic + propionate	3.43	0.50	14.00	169
Butyric + butyrate	3.18	0.55	15.00	170
Combination of them	3.16	0.54	14.60	172
SE	± 0.43	± 0.03	± 0.41	± 6.31
Significances	NS	NS	NS	NS

At the 0.05 level of probability, means that have the same letter inside the same column are not statistically different; NS stands for not significant (*P* > 0.05).

Total blood protein, ALT, creatinine, and AST levels were statistically unchanged (*p* > 0.05) across all dietary interventions, suggesting that OA + S had no detrimental impact on liver or kidney function.

### Thyroid hormones

The influence of OA + S on thyroid hormones is seen in Table 4.

**Table 4:** Demonstrates the impact of dietary modifications on thyroid hormones at 35 days of age.

Item	T3 (ng/ml)	T4 (ng/ml)	T4/T3
Control	4.53	13.84^b^	3.06
Lactic + lactate	5.05	16.08^a^	3.19
Propionic + propionate	4.83	15.85^a^	3.29
Butyric + butyrate	5.08	16.45^a^	3.24
Combination of them	4.91	16.29	3.32
SE	± 0.15	± 0.31	± 0.05
Significances	NS	**	NS

At the 0.05 level of probability, means that have the same letter inside the same column are not statistically different; **P < 0.01, NS: Not significant (*P* > 0.05).

No discernible changes (*p* > 0.05) in the amount of T3 or T4/T3 ratios. All the groups that received supplements had significantly increased (*p* ≤ 0.05) thyroxine (T4) levels than the control group, showing that the dietary changes raised thyroxine levels without changing the balance of thyroid hormones.

### Antioxidant status

**Fig. 1:**
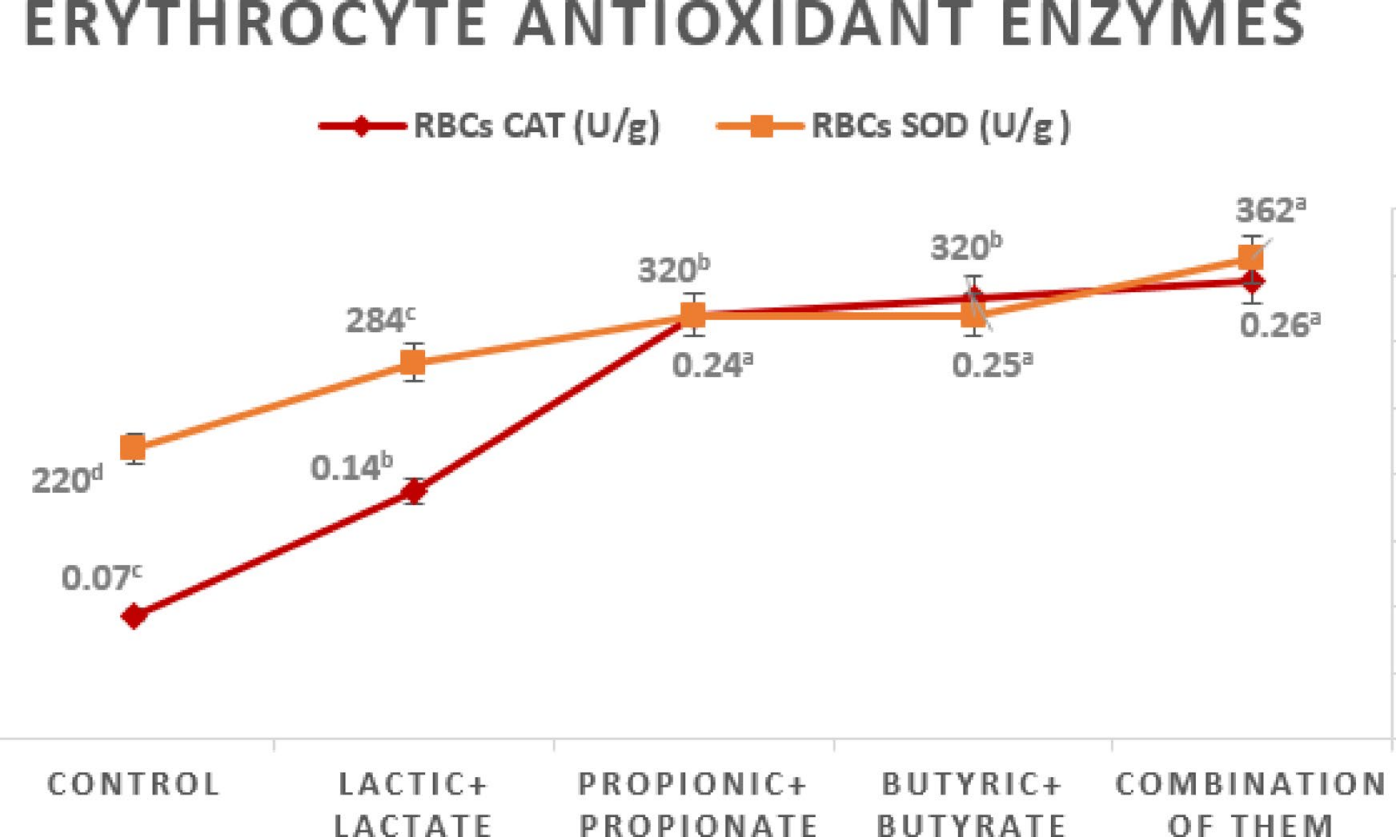
The impact of dietary interventions on the activation of serum antioxidant enzymes in red blood cells at 35 days of age.

**Fig. 2:**
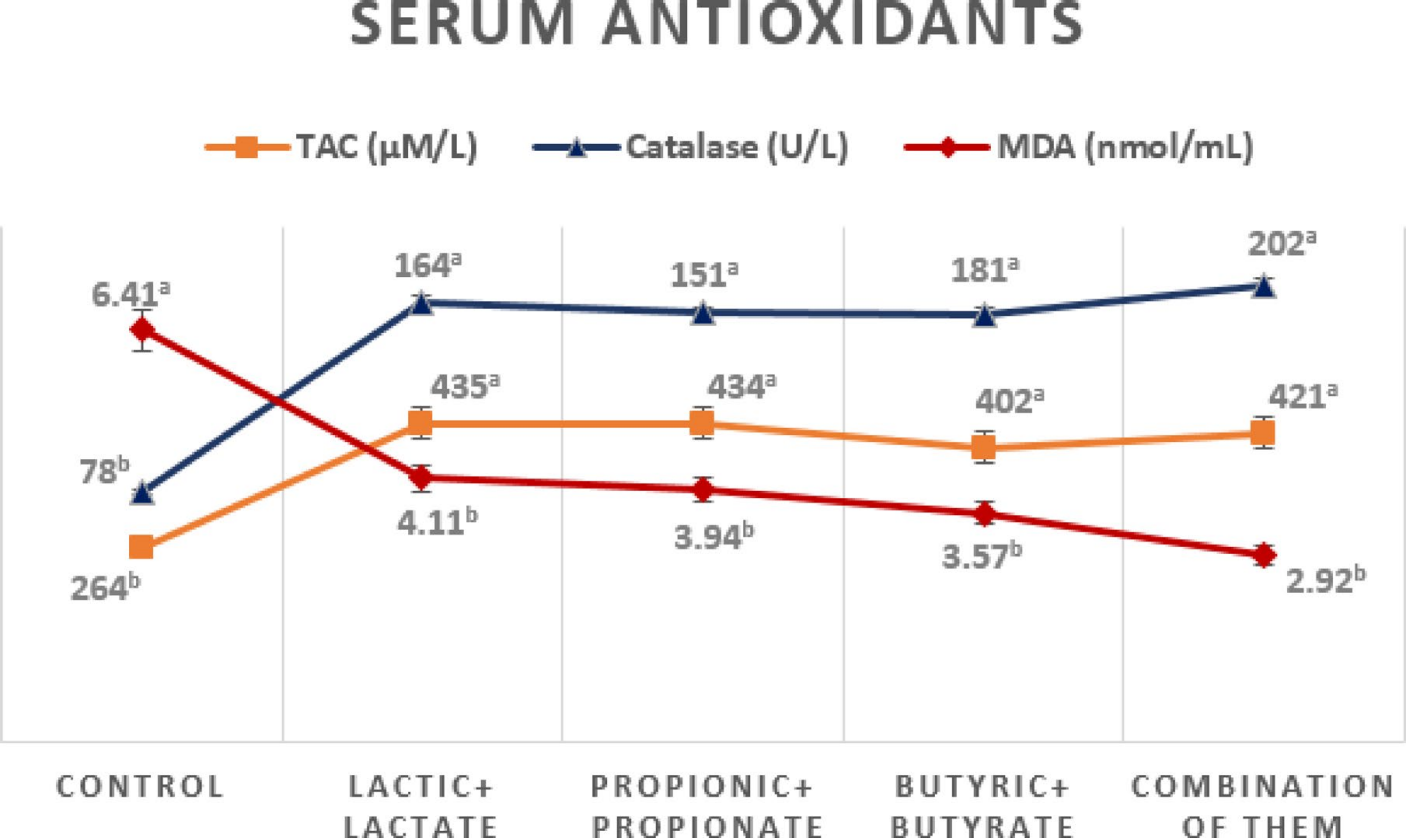
The impact of dietary treatments on redox balance at 35 days of age.

The impact of OA + S on antioxidant parameters in red blood cells (RBCs) and serum are presented in Figs. 1 and 2.

Supplementation with OA + S significantly enhanced (*p* ≤ 0.05) the activities of catalase (CAT) and superoxide dismutase (SOD) in RBCs. Furthermore, birds fed diets containing propionic or butyric acids and their salts, or a combination of three organic acid salts, showed the highest (*p* ≤ 0.05) CAT activity (about 25 U/g), whereas the control group only had 0.07 U/g. When birds were given a combination of three organic acid salts, their highest SOD activity (*p* ≤ 0.05) was 362 U/g, while the control group’s was 220 U/g.

In serum, the MDA level significantly (*p* ≤ 0.05) decreased (about 3.6 nmol/mL) given diets containing organic acids and their salts compared to the control (6.4 nmol/mL). Moreover, TAC (about 423 µM/L) and CAT activity (about 174 U/L) were significantly higher (*p* ≤ 0.05) compared with the control values (264 µM/L and 78 U/L, respectively). These results indicate an enhanced redox balance by increasing antioxidant enzymes and reducing oxidative stress.

### Insulin-like growth factor

**Fig. 3:**
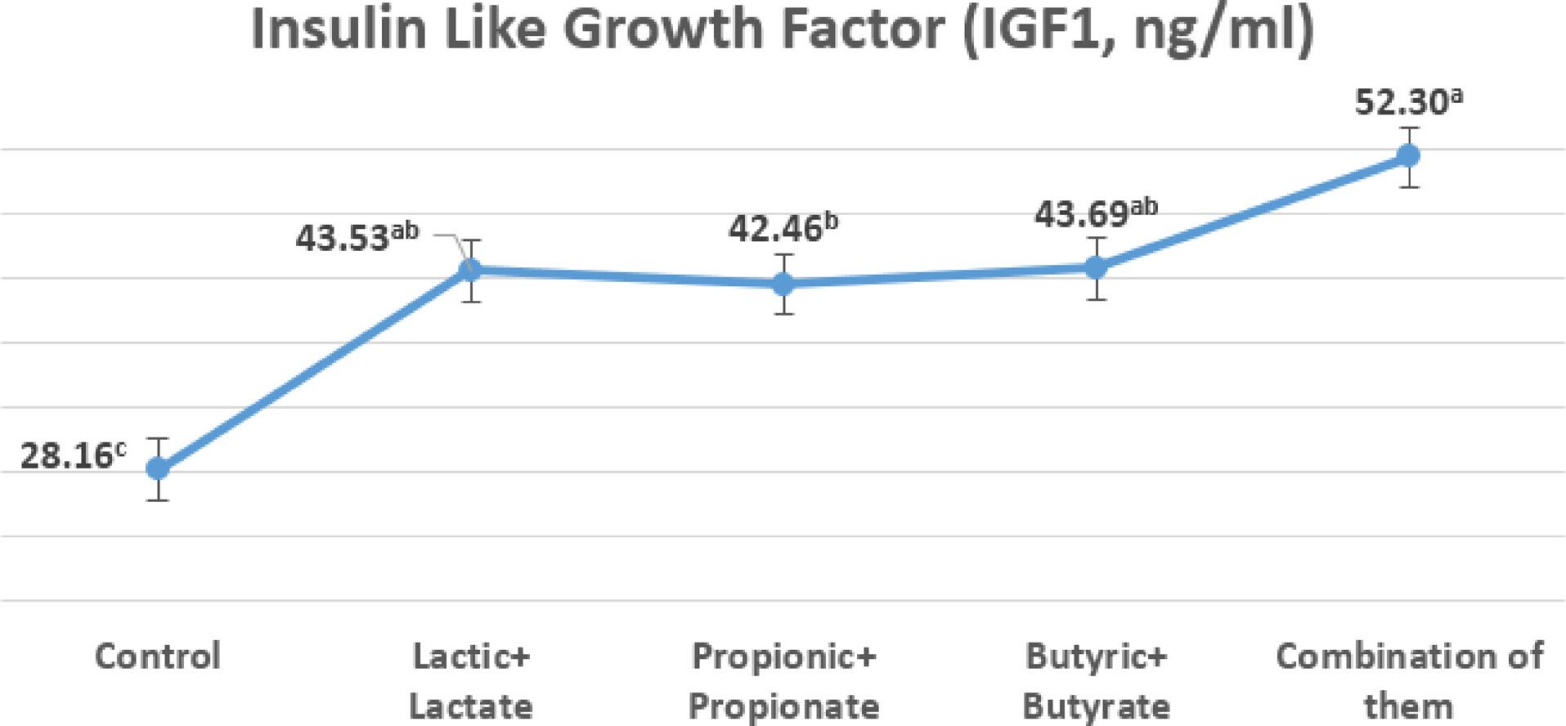
The impacts of dietary treatments on IGF1 at 35 days of age.

Figure 3 illustrates the impact of OA + S on insulin-like growth factor.

At the end of the experiment, serum IGF-1 levels in all treatments significantly (*p* ≤ 0.05) increased following dietary treatments containing OA + S (about 45.5 ng/ml) in contrasting to the control (28.16 ng/ml).

### Carcass characteristics

Table 5 illustrates the influence of OA + S on carcass properties.

**Table 5:** Dietary treatments’ effects on carcass characteristics at 35 days of age.

Item	Dressing (%)	Liver (%)	Gizzard (%)	Heart (%)	Spleen (%)	Bursa (%)	Intestinal length (Cm)
Control	68.52^b^	2.56	1.71	0.65	0.09	0.17	162^b^
Lactic + lactate	73.04^a^	2.89	1.68	0.53	0.13	0.23	195^a^
Propionic + propionate	72.44^a^	2.44	1.45	0.50	0.08	0.17	185^a^
Butyric + butyrate	73.66^a^	2.64	1.62	0.56	0.10	0.18	192^a^
Combination of them	75.08^a^	2.46	1.61	0.55	0.11	0.17	190^a^
SE	± 2.22	± 0.09	± 0.27	± 0.04	± 0.02	± 0.01	± 10.07
Significances	**	NS	NS	NS	NS	NS	*

At the 0.05 level of probability, means that have the same letter inside the same column are not statistically different; *P < 0.05,** *P* < 0.01, NS: Not significant (*P* > 0.05).

All groups fed diets contained OA + S demonstrated significant improvements (*p* ≤ 0.05) in dressing (approximately 73.6%) and intestinal length (approximately 190.5 cm) when compared to control (68.52% and 162 cm, respectively), while no significant differences (*p* > 0.05) across all treatments in comparative weights of the gizzard, liver, heart, spleen, and bursa of Fabricius. It points to a tendency for higher carcass output.

### Intestinal microbiota

**Fig. 4:**
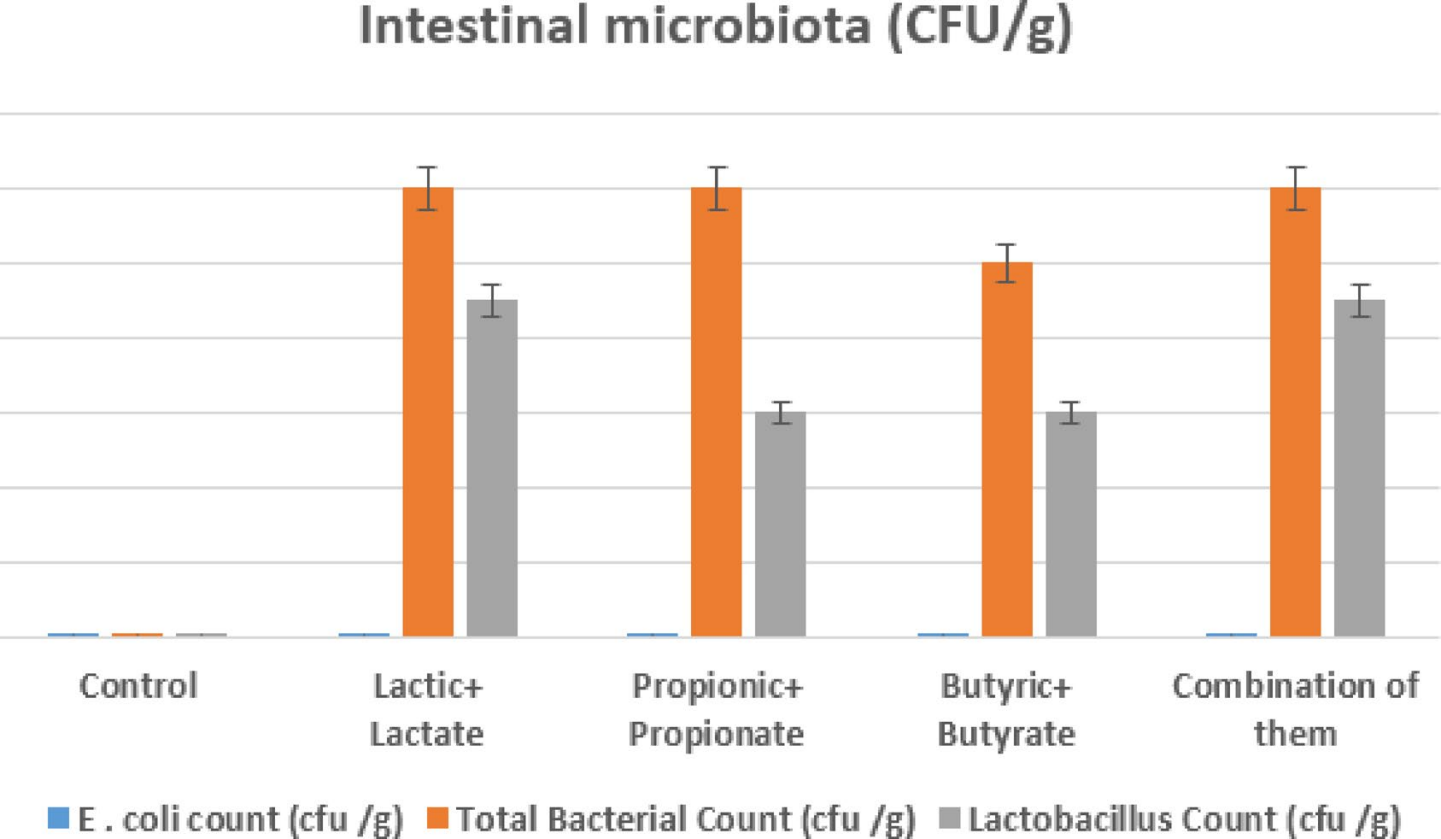
The impact of dietary modifications on the gut microbiota at 35 days of age.

Figure 4 illustrates the influence of OA + S on gut microbiota.

All supplemented groups showed a significant rise in *Lactobacillus* spp. counts, along with an increase in overall bacterial counts. Crucially, no *Escherichia coli* was found in any of the groups that received treatment, suggesting that organic acids have strong antibacterial properties because they increase useful bacteria and decrease harmful bacteria.

### Immune response

**Fig. 5:**
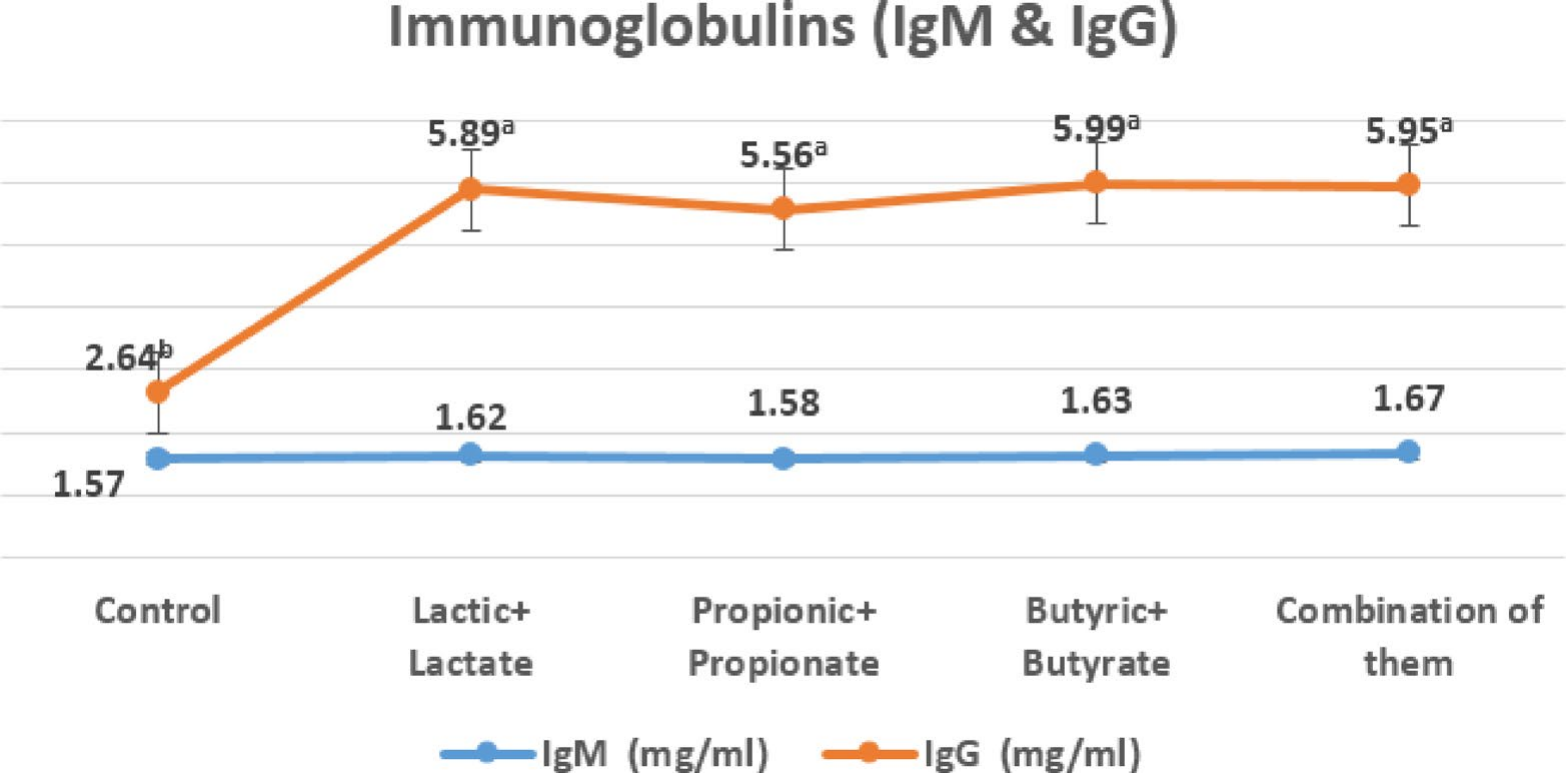
The effect of dietary treatments on IgM and IgG at 35 days of age.

At 35 days of age, nutritional supplementation with organic acids and their salts markedly affected IgG concentrations, although no significant impact was noted on IgM levels, as indicated in Fig. 5.

All treatments had significantly improved in IgG (*p* ≤ 0.05) than the control, with values between 5.56 and 5.99 mg/mL compared to 2.64 mg/mL in the control. Conversely, There were no discernible variations in IgM levels across the groups (*p* > 0.05), with concentrations ranging from 1.58 to 1.67 mg/mL versus 1.57 mg/mL in the control, all within the anticipated physiological range for 35-day-old broilers under non-stress conditions.

**Fig. 6:**
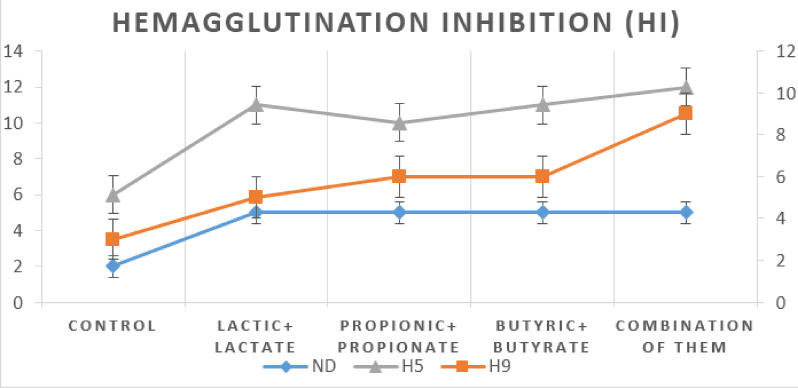
The effect of dietary treatments on the hemagglutination inhibition at 35 days of age.

Figure 6 demonstrates the effects of OA + S on hemagglutination inhibition.

All treatment groups showed noticeably higher hemagglutination inhibition (HI) titers against NDV, H9, and H5 than the control. The combination group exhibited the greatest immune responses, particularly against H9 and H5, achieving titers of 9 and 10, respectively.

## Discussion

This study assessed the impact of feed supplementation with lactic, propionic, and butyric acids and their salts on growth performance, metabolic hormones, redox balance, immunity, and intestinal microbiota in broilers. The findings indicated that these additives obtained several advantages in physiological responses and productivity without any negative effects on liver or kidney function.

### Growth performance

The observed improvements in BWG and FCR during the grower, finisher, and overall periods indicate that organic acids enhance gastrointestinal function and nutrient utilization. The superior performance observed with the combined supplementation of organic acids and their salts may be attributed to the synergistic effects of acidifiers and their salts acting across different sections of the gastrointestinal tract. Previous studies have reported that organic acids reduce gastrointestinal pH, thereby stimulating digestive enzyme activity, suppressing pathogenic bacteria, and promoting beneficial microbiota, ultimately improving digestive efficiency^14–21^.

From a practical perspective, these increases in BWG and FCR not only result in increased growth efficiency but also lower feed costs and support sustainability in systems used for the production of poultry^32^.

Organic acids and their salts are widely recognized as effective alternatives to antibiotic growth promoters in poultry production. As weak acids containing a carboxylic acid group, they serve as intermediates in the metabolism of carbohydrates, proteins, and fatty acids^33^. Propionic acid, in particular, has demonstrated potential to improve poultry health and meat quality by enhancing nutrient utilization and overall broiler performance^34^. Similarly, sodium butyrate supplementation at 1% has been shown to improve growth and nutrient absorption in broilers^35^, while other studies confirmed that organic acid inclusion in diets consistently enhances body weight gain and health^12,36^.

From a practical perspective, these increases in BWG and FCR not only result in increased growth efficiency but also lower feed costs and support sustainability in systems used for the production of poultry^37,38^.

### Blood biochemical parameters

Serum levels of creatinine, ALT, AST, and total protein did not differ significantly across the experimental groups. This result suggests that hepatic or renal activity was unaffected by feed supplementation with organic acids and their salts. These results align with prior studies by Elnaggar and El-kelawy^39^ and Masiuk^40^, which revealed that broilers given diets containing organic acid mixes had homeostatic biochemical profiles without any adverse effects.

### Thyroid hormones

Although there were no changes in triiodothyronine (T3) or the T3/T4 ratio, the treated groups’ significant elevation of serum thyroxine (T4) in the current study suggests that the dietary intervention primarily increased thyroidal secretion of T4 rather than influencing the peripheral deiodination of T4 into T3^41^. Thyroxine (T4) is the main hormone secreted by the thyroid gland and functions primarily as a prohormone, whereas triiodothyronine (T3) is the physiologically active metabolite produced by extrathyroidal deiodination by type I and type II deiodinases^42^. A rise in T4 may indicate better physiological function and metabolism, as it is a crucial hormone for controlling development and basal metabolic rate^43^.

Under normal physiological conditions, deiodinase activity may have stayed constant (homeostatic mechanisms), preserving euthyroid state and avoiding excessive metabolic stimulation, as indicated by the steady levels of T3 and the unaltered T3/T4 ratio^44^. These findings suggest that dietary organic acids enhanced thyroidal activity while maintaining systemic hormonal balance, thereby supporting improved growth physiology.

### Antioxidant status

The substantial decrease in MDA and the improvement of both enzymatic and non-enzymatic antioxidant indicators refer to the strong antioxidant properties of organic acids. Because of their high metabolic demands, broilers that grow quickly are particularly vulnerable to oxidative stress. Perhaps as a result of the combined impacts of many pathways, such as enhanced nutrient absorption, direct scavenging activity, and microbial regulation, the supplementation of acids and their salts offered the strongest antioxidant protection^15–22,32–40,43–45^.

### Insulin like growth factor

The notable rise in IGF-1 levels among treated groups is consistent with Pearlin et al.^13^, who reported enhanced IGF-1 expression in broilers fed organic acids, most likely due to improved gut integrity and nutrient absorption. Beyond this nutritional effect, thyroid hormones particularly T4 stimulate hepatic IGF-1 production by increasing liver sensitivity to growth hormone (GH). IGF-1 is a crucial anabolic mediator that promotes protein accretion, skeletal development, and overall somatic growth in chickens^46^.

The contemporaneous elevation of T4 and IGF-1 in the present study supports this mechanistic connection, aligning with earlier reports that characterized thyroid hormones as modulators of growth efficiency and IGF-1 expression^47–49^.

When considered collectively, the observed increase in T4 and the improvement in IGF-1 suggest a synergistic endocrine effect of organic acid supplementation. Organic acids and their salts appear to enhance both the thyroidal and somatotropic axes by improving nutrient absorption, redox balance, and endocrine responsiveness. This may help to explain the superior growth performance and physiological resilience observed in the treated broilers^50^.

### Carcass characteristics

Numerical trends showed improved carcass yield in the treated groups, even if the observed improvements in organ weights were not significant (*p* >0.05). These findings agree with previous studies that demonstrate the ability of organic acids to affect fat metabolism and encourage the formation of lean tissue^51,52^.

### Intestinal microbiota

Organic acids have antibacterial properties, especially their ability to lower pH, inhibit harmful bacteria, and improve useful bacteria, as demonstrated by the almost total suppression of *E. coli* and the rise in *Lactobacillus* species. Such gut microbiota modification fosters an environment that is conducive to immunological development and nutrient digestion. Studies using acidifiers as antibiotic substitutes have shown similar microbiological patterns^14–22,27,33–45^. A vast array of chemical substances that are frequently found in nature as essential components of plant, livestock and microbe tissues are referred to as organic acids. By lowering the digestive tract’s pH, organic acids encourage the growth of useful bacteria^22^. Organic acids’ antibacterial, antifungal, antiprotozoal, and anticoccidial properties have made them a promising alternative to antibiotics^12^.

### Humoral immunity

This study found that broilers raised in non-stressful environments maintained consistent IgM concentrations throughout all groups, with no significant (*p* >0.05) differences recorded. These findings indicate that although organic acid supplementation may improve certain immunological parameters, such as IgG, it does not substantially influence IgM production at 35 days of age under normal health conditions. The absence of variation in IgM may result from the birds existing in a non-challenged condition, wherein the baseline immunoglobulin synthesis is adequate to sustain homeostasis. The observed increase in IgG levels across all treatment groups signifies an augmented humoral immune response. This improvement can be ascribed to the immunostimulatory properties of organic acids, which enhance gut microbiota equilibrium and mucosal immunity^53^. The dietary combination of organic acids likely conferred synergistic advantages, augmenting the systemic immune response. The lack of substantial alterations in IgM levels aligns with research indicating that primary immunological responses, as indicated by IgM, exhibit less sensitivity to dietary modifications in non-stressful environments^14^. The consistent IgM levels fall within the usual range established for healthy broilers of 35 days of age, suggesting an absence of immunosuppression or infection throughout the trial^27^.

Increased humoral immune responses are indicated by the rise of HI titers, particularly against H9 and H5. By preserving intestinal integrity, enhancing nutritional availability, and modifying immunological signaling pathways, organic acids may help immune function. Rahman et al.^8^ and Biagini et al.^54^ have similarly shown increased antibody responses in broilers fed organic acid supplements in diets. Organic acids augment nutrient digestion and bolster immunity in chickens, hence obviating the need for antibiotics^22^. Organic acids protect the gastrointestinal tract (GIT) by improving its physiology^12^. The power of OA, either by themselves or in combination, to enhance the broiler’s defenses against ND, H5, H9, and SRBCs^36^.

### Mode of action of OA + S in broiler diets

Organic acids (like lactic, butyric and propionic acids) and their salts (like sodium lactate, propionate and butyrate) are promising poultry feed additives as growth promoters. These acids and salts have various beneficial effects on gut health and function.

Organic acids primarily function by lowering the gastrointestinal system pH, especially in the proventriculus and gizzard, to promote the activity of digestive enzymes and reduce harmful bacteria such as *E. coli*. and *Salmonella Sp.*^55,56^. Additionally, they enhance growth performance and nutrient digestibility^37^.

On the other hand, organic acid salts are more stable during feed processing and less volatile than free acids, making them better suited for producing of pelleted feed^57,58^. These salts play a role in gut health and microbial balance by releasing their acid component gradually in the lower intestine, which permits antimicrobial effects to spread deeper into the gastrointestinal tract^20,53–59^. The free acids mainly operate in the gizzard and proventriculus, the top portion of the digestive system, in contrast to the salts that, after dissociation, become active deeper down in the gut and have a longer-lasting effect.

Furthermore, in addition to their antibacterial qualities, organic acids and their salts have other physiological and immunological advantages^19^. By improving mineral solubility (Ca, P, Zn), a decrease in stomach pH promotes nutrient absorption and epithelium growth^60^. The main energy source for colonocytes is butyric acid, which also promotes the elongation of villus height, decreases crypt depth, and increases tight junction proteins like occludin and claudin, which preserve the integrity of the mucosal barrier^61^. While lactic acid prevents enteric pathogens from adhering, it also encourages the growth of good bacteria such as Lactobacillus species^62^. In parallel, salts like sodium butyrate and others help maintain immunological homeostasis by reducing NF-κB activation, which in turn modulates inflammatory responses^63^. Overall, these procedures improve BWG and FCR, offering a more environmentally friendly and antibiotic-free way to produce broilers in the modern era^64^.

### General consideration

The results demonstrate their potential in systems for producing broiler chicken without the use of antibiotics and provide support to the concept of acidifier synergism.

The combination of organic acids and their salts consistently enhance productive performance across all evaluated parameters. In addition, it raises the possibility that combining many organic acids could have wider physiological and antibacterial advantages.

### Research limitations

This study provides valuable insights into the physiological and immunological effects of organic acids and their salts on broiler health and performance. However, it is important to acknowledge that certain inherent experimental limitations may exist, as is typical in controlled animal studies. The experiment was carefully designed to minimize variability and ensure the reliability of the collected data; nonetheless, further investigations conducted under diverse practical and production conditions may offer complementary perspectives and enhance the broader applicability of the present findings.

## Conclusion

Broiler diets can be supplemented with organic acids, including lactic, propionic, or butyric acids, and their salts, instead of antibiotic growth promoters. These additives improved growth performance, feed conversion, antioxidant activity, thyroid function, and humoral immunity. Additionally, these additives enhance gut microbial balance by suppressing harmful bacteria and promoting beneficial ones, all without adverse effects on liver or kidney function.

Future studies could look more closely at exploring other types of organic acids with their salts, synergistic processes, and long-term impacts on meat quality.

## Data Availability

Requests for materials and correspondence should be sent to A. Samy at [Asamy1@yahoo.com](mailto: Asamy1@yahoo.com) .
